# The pleiotropic vasoprotective functions of high density lipoproteins (HDL)

**DOI:** 10.7555/JBR.31.20160103

**Published:** 2017-01-15

**Authors:** Guilaine Boyce, Emily Button, Sonja Soo, Cheryl Wellington

**Affiliations:** Department of Pathology and Laboratory Medicine, Djavad Mowafaghian Centre for Brain Health, University of British Columbia, Vancouver, BC V6T 1Z3, Canada.; Department of Pathology and Laboratory Medicine, Djavad Mowafaghian Centre for Brain Health, University of British Columbia, Vancouver, BC V6T 1Z3, Canada.; Department of Pathology and Laboratory Medicine, Djavad Mowafaghian Centre for Brain Health, University of British Columbia, Vancouver, BC V6T 1Z3, Canada.; Department of Pathology and Laboratory Medicine, Djavad Mowafaghian Centre for Brain Health, University of British Columbia, Vancouver, BC V6T 1Z3, Canada.

**Keywords:** high density lipoprotein, vascular function, vascular disease, alzheimer disease, HDL-proteome, HDL-lipidome

## Abstract

The pleiotropic functions of circulating high density lipoprotein (HDL) on peripheral vascular health are well established. HDL plays a pivotal role in reverse cholesterol transport and is also known to suppress inflammation, endothelial activation and apoptosis in peripheral vessels. Although not expressed in the central nervous system, HDL has nevertheless emerged as a potential resilience factor for dementia in multiple epidemiological studies. Animal model data specifically support a role for HDL in attenuating the accumulation of β-amyloid within cerebral vessels concomitant with reduced neuroinflammation and improved cognitive performance. As the vascular contributions to dementia are increasingly appreciated, this review seeks to summarize recent literature focused on the vasoprotective properties of HDL that may extend to cerebral vessels, discuss potential roles of HDL in dementia relative to brain-derived lipoproteins, identify gaps in current knowledge, and highlight new opportunities for research and discovery.

## The pleiotropic functions of high density lipoprotein (HDL)

**Fig.1:**
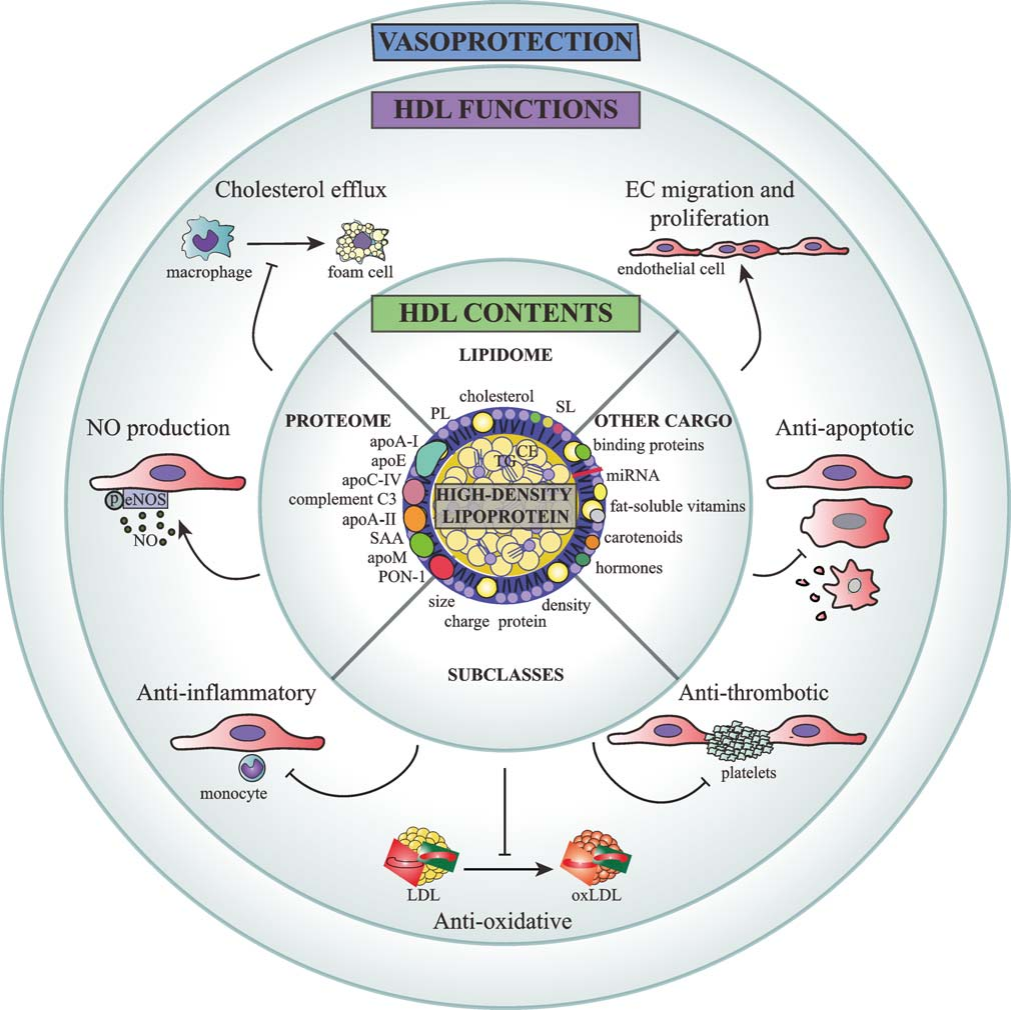
Pleiotropic contents and functions of HDL. apoA-I: apolipoprotein A-I; apoA-II: apolipoprotein A-II; apoC-IV: apolipoprotein C-IV; apoE: apolipoprotein E; apoM: apolipoprotein M; EC: endothelial cell; eNOS: endothelial nitric oxide synthase; LDL: low density lipoprotein; miR-223: micro RNA 223; NO: nitric oxide; oxLDL: oxidized LDL; p: phosphate group; PL: phospholipid; PON-1: paraoxonase 1; S1P: sphingosine-1-phosphate; SAA: serum amyloid A; SM: sphingomyelin.

Like other mature lipoproteins, HDL consists of a core of hydrophobic lipids surrounded by a phospholipid and free cholesterol monolayer studded by proteins (***Fig. 1***)^[1]^. A key protein found in most HDL particles is apolipoprotein A-I (apoA-I), which makes up 70% of its protein content^[2]^. The major lipid classes found on HDL include cholesterol and other steroids, phospholipids, cholesteryl esters, sphingolipids, and triglycerides^[3]^. Overall, HDL particles consist of approximately 85-95 distinct proteins^[4]^ and hundreds of lipid subtypes^[5]^ that together mediate diverse functions including lipid transport and metabolism, anti-oxidation, immune response, hemostasis, metal binding, and vitamin transport^[5^–^7]^.

HDL is the smallest and densest of the plasma lipoproteins and contains an estimated 85-95 distinct proteins, 200 lipid species and several other nonpolar cargo molecules. HDL components and subclass distribution can vary between individuals and is altered by diseased states. The compositional profile of HDL confers pleiotropic functions to the population of circulating particles.

## HDL-C and cardiovascular disease

The association between low HDL cholesterol (HDL-C) levels and elevated cardiovascular disease (CVD) was first suggested in the 1960s in the Framingham Heart Study^[8]^. Since then, a multitude of clinical studies have strengthened this relationship^[9^–^11]^. Although Mendelian randomization studies have now demonstrated that HDL-C levels per se have no causal relationship with CVD^[12^–^14]^, the question remains as to whether HDL-C, a static measure of HDL's cholesterol content, adequately reflects the beneficial functions of HDL on vascular health.

In humans, genetic deficiency of *APOA-I* or the ATP binding cassette transporter 1 (*ABCA1*) leads to very low levels of HDL-C that can be associated with increased risk of and accelerated onset of coronary artery disease (CAD)^[15]^. Similar outcomes are observed in some but not all cases of lecithin cholesterol acyltransferase (*LCAT*) deficiency^[15]^. However, other forms of genetically altered HDL-C levels suggest that there is more complexity to the role of HDL in health and disease. For example, carriers of the *APOA-I* mutation known as apoA-I Milano have very low HDL-C levels but similar levels of atherosclerosis and CAD as controls with normal HDL-C levels^[16]^. Conversely, carriers of mutations in *SCARB1*, the gene encoding HDL receptor scavenger receptor class B type I (SR-B1), have abnormally high HDL-C levels and yet are at increased risk for CAD^[17]^.

Unlike humans where the major plasma lipoprotein is low-density lipoprotein (LDL), the major plasma lipoprotein in mice is HDL. The innately high HDL:LDL ratio in mice renders them generally resistant to CVD and advanced atherosclerosis compared to humans^[18]^. As a result, atherosclerosis studies in mice overwhelmingly depend on genetically modified models, including deficiency of either apoE or low-density lipoprotein receptor (LDLR), to allow the effects of HDL on atherosclerosis to be studied^[19]^. For example, genetic deletion of either *apoA-I*, *ABCA1*, *LCAT* and *SR-B1* in *apoE* ^−/−^ or *LDLR* ^−/−^ animals alters murine HDL-C levels. In addition, the effects on atherosclerosis can also vary with strain background and animal diet^[20]^. Nonetheless, HDL-targeted therapies in murine models of atherosclerosis appear, overall, to be beneficial. For example, transgenic overexpression of apoA-I, gene transfer of human apoA-I, adenoviral transfer of apoA-I, and infusion with recombinant apoA-I or HDL can reduce or stabilize atherosclerotic plaques in *apoE* ^−/−^ or *LDLR* ^−/−^ mice^[21]^.

## Changes in HDL cholesterol efflux capacity

The cholesterol efflux capacity (CEC) of HDL is modified in CVD^[22^–^26]^, metabolic syndrome^[27]^, and during acute inflammation^[28]^. How distinct CEC and HDL-C are as biomarkers of disease has been debated, with some studies observing reduced CEC independent of changes in HDL-C^[22^-^23^,^25^,^28^–^30]^, while others find that CEC and HDL-C changes correlate^[24^,^26^-^27]^. Possible mechanisms to explain diminished CEC in most of these studies is increased HDL-associated serum amyloid A (SAA)^[28]^ and reduced HDL-associated paraoxonase 1 (PON-1)^[26]^ in inflammatory states, which will be discussed below. Importantly, despite its obvious implication for reverse cholesterol transport (RCT), CEC is not the only known function of HDL. Here, we will discuss additional vasoprotective properties of HDL, with a focus on HDL's anti-inflammatory, anti-oxidative, vasodilatory and anti-apoptotic functions.

## Anti-inflammatory effects of HDL

Several mechanisms by which HDL exerts anti-inflammatory effects on endothelial cells have been described. These include pathways dependent on the HDL receptor SR-B1^[31^–^33]^ as well as through vascular sphingosine 1 phosphate (S1P) receptor 1 and 3^[34]^, which trigger a signaling cascade through the PI3K/Akt pathway leading to phosphorylation of endothelial nitric oxide synthase (eNOS). The vasoprotective effects of nitric oxide produced by eNOS phosphorylation are well-established, and include vasodilation, reduced endothelial cell permeability, and inhibition of vascular cell adhesion molecule-1 (VCAM-1) expression via downregulation of the pro-inflammatory NFκB signaling pathway^[35]^. HDL-S1P action can also directly inhibit NFκB signaling to suppress adhesion molecule expression^[34]^, reduce endothelial exocytosis^[31]^, and maintain annexin-1 expression^[33]^. Additionally, HDL can indirectly increase eNOS activity via actions of the lipid transporter ABCG1 to maintain proper membrane fluidity for eNOS function^[36^,^37]^. The HDL-associated protein PON-1 prevents lipid and LDL oxidation, thereby protecting endothelial cells from oxidative damage, pro-inflammatory signaling, and apoptosis^[38^-^39]^.

Many disease states, particularly those with an inflammatory component, can affect HDL's vasoprotective functions (***Table 1***). For example, HDL isolated from CVD patients exhibits reduced ability to phosphorylate eNOS^[6^,^40^-^41]^ and displays a distinct repertoire of immune cell trafficking proteins^[41]^. HDL isolated from children with chronic kidney disease exhibits reduced ability to protect from endothelial cell activation ^[42^-^43]^. Acute inflammation, as in the case of periodontal therapy, can also alter the ability of HDL to induce eNOS phosphorylation^[44]^. In abdominal aortic aneurysm (AAA), a quantitative reduction in apoA-I-mediated vasoprotection may result from decreased levels of circulating small HDL, a process which itself may partially be due to the sequestering of apoA-I at the site of inflammation in thrombotic aortic tissue^[45^-^46]^. Moreover, inflammatory-remodelling of HDL composition during AAA by the inclusion of pro-oxidant proteins may further reduce HDL quality and contribute to the observed loss of anti-thrombotic and anti-oxidative capacity^[46]^. In contrast, exercise training improves the ability of HDL to protect endothelial cells from tumor necrosis factor-α induced injury, monocyte adhesion, and VCAM-1 expression in metabolic syndrome while also elevating eNOS activation^[47]^. Importantly, changes to the anti-inflammatory functions of HDL have even been observed in disease in the absence of changes in circulating HDL-C levels^[43^-^44]^.

**Tab.1:** HDL compositional and functional heterogeneity in disease

Disease	Proteome	Lipidome	Size	Function
Cardiovascular	↓/--PON-1[^26^,^63^,^71^]	↓S1P[^90^], SM[^240^]	↓/↑ large HDL[^23^,^59^-^61^,^64^]	↓ cholesterol efflux[^25^,^26^]
disease	↑ SAA[^41^, ^84^, ^239^]	↑/↓PL[^239^-^241^]	↓ small HDL[^63^]	↓ eNOS phosphorylation[^23^,^41^,^75^]
	↓ apoA-I, apoA-II, apoE[^84^]	↑TG[^239^,^240^]		↑ inflammatory activity[^242^]
Acute inflammation	↓ PON-1[^28^,^44^,^74^] ↑ SAA, apoA-II, complement C3[^44^,^74^] ↓/↑apoA-I[^44^,^74^]	↓S1P, SM[^74^,^243^] ↑TG, FFA[^243^]		↓/-- cholesterol efflux[^28^,^44^,^74^] ↓ NO production[^44^,^74^] ↑ inflammatory activity[^44^] ↑ oxidative activity[^44^]
Chronic kidney disease	↓/--PON-1[^76^,^77^,^82^] ↑SAA, SDMA, apoC-II[^43^,^75^,^81^,^98^] ↓ apoA-I, apoA-II[^75^,^81^]	↑ TG[^81^] ↓ PL[^81^]	↓ small HDL[^244^]	↓ NO production[^43^] ↓ cholesterol efflux[^43^,^81^] ↑ inflammatory activity[^42^,^43^] ↑ oxidative activity[^43^,^98^] ↓ EC proliferation and migration[^42^]
Cirrhosis	↓/↑ PON-1[^58^,^73^] ↓ apoA-I, -II, apoC-II, -III[^72^] ↑ SAA, apoE[^72^]	↑ PL[^73^]	↑ large HDL[^72^,^73^]	↓ cholesterol efflux[^72^] ↓ PON1 activity[^73^]
Aging	-- PON-1[^78^,^79^] ↓ apoE[^78^] ↑ SAA, complement C3[^78^]	↑ SM[^245^]		↑ oxidative activity[^78^,^79^] ↓/-- cholesterol efflux[^78^,^245^] ↓ PON1 activity[^78^,^79^]
Arthritis	↓ PON-1[^77^] ↑ SAA[^77^]	↑ SM, PL[^246^]		↑ inflammatory activity[^247^]
Type 2 diabetes mellitus	↓ apoA-I[^85^] ↑ apoA-II[^85^]	↑TG, S1P[^85^,^90^,^248^]		-- cholesterol efflux[^27^] ↓ NO production[^27^] ↑ inflammatory activity[^248^]
Alzheimer's disease	?	?	?	↓ cholesterol efflux[^249^] ↑ inflammatory activity[^249^]
Age-related macular degeneration	↑ SAA[^250^]	?	?	↑ anti-inflammatory activity[^250^]

apoA-I: apolipoprotein A-I; apoA-II: apolipoprotein A-II; apoC-II: apolipoprotein C-II; apoC-III: apolipoprotein C-III; apoE: apolipoprotein E; EC: endothelial cell; eNOS: endothelial nitric oxide synthase; FFA: free fatty acid; NO: nitric oxide; PL: phospholipid; PON-1: paraoxonase 1; S1P: sphingosine-1-phosphate; SAA: serum amyloid A; SM: sphingomyelin; SMDA: symmetrical dimethylarginine; TG: triglyceride; ↓: decrease; ↑: increase; --: no change; ?: unknown in literature.

## HDL-C is an imperfect marker

The disassociation between HDL *functions* and HDL-C *levels* may help to explain why the epidemiological associations of HDL and CVD risk are not easily captured by mere HDL-C measures. HDL functional assays may also be more informative than HDL-C measures in clinical trials. For example, efforts to use statins, niacin, and cholesteryl ester transfer protein (CETP) inhibitors to raise HDL-C levels and protect against CVD have been disappointing^[48^–^50]^. Although statins consistently reduce CVD events, the prognostic utility of HDL-C in statin users is unclear^[51^–^54]^. Two large randomized control trials to test the effect of niacin on CVD showed no statistically significant reduction in CVD despite elevated HDL-C levels^[42^,^55^–^57]^. CETP inhibitors also failed to reduce CVD events despite raised HDL-C levels^[49]^. A meta-analysis of randomized controlled trials for niacin, fibrates, and CETP inhibitor therapies in conjunction with statin therapy found no change to mortality, CAD mortality and myocardial infarction compared to patients treated with statins alone^[58]^.

## HDL heterogeneity and modification in disease

HDL can be classified by a variety of schemes including apolipoprotein content, size, surface charge, and density (***Fig. 1***)^[2]^. The distribution of HDL-C among different sizes has been observed to vary with exercise, CVD risk factors, and CVD disease status and lipid-lowering medications. However, the direction of association between HDL subclass and disease outcomes has been controversial. Many studies have found that large HDL subclasses appear to be beneficial for cardiovascular health with stronger associations with disease than total HDL-C^[23^,^59^–^62]^. By contrast, other studies have observed that CAD patients have lower levels of small HDL^[63]^ and elevated levels of large HDL^[64]^. Investigations on the effect of statins on HDL subclass is also not as clearly defined as their well-established ability to elevate total HDL-C levels^[65]^. For example, statins have been found to exert no effect^[66]^ or to lead to elevated levels of the large HDL2 subclass while decreasing levels of small HDL3 levels^[67^-^68]^, although contrasting reports indicate an increase in HDL3 levels^[69]^. Fibrates increase HDL3 levels while decreasing those of HDL2^[65]^ whereas niacin has the opposite effect on HDL subclass by promoting conversion to mature HDL2 particles^[65]^. Combination treatments of lipid lowering drugs present no net change in HDL subclass compared to monotherapy^[67]^ or have reported an additive effect with improved HDL functionality^[70]^. Variations in cohort, drug regimen and experimental techniques likely contribute to the varying observations of medication use on HDL subclass distribution and net function.

To add further complexity to the matter of HDL subclass, a recent report has found that HDL appears to be secreted from the liver in all of its unique sizes and remains in those size classes for several days before excretion^[71]^. This is contrary to the traditional view of how HDL subclasses are formed, which posits that HDL is first secreted from the liver as small, lipid-poor, discoidal HDL that is lipidated to evolve into the larger spherical forms over time.

Another measure of interest is the heterogeneity of the HDL proteome. The HDL proteome varies considerably between individuals based on disease, diet, age, and inflammatory status (Table 1). For example, PON-1 content or activity on HDL is reduced in patients with CVD^[26^,^72]^ liver cirrhosis^[73^-^74]^, acute inflammation^[28^,^44^,^75]^, chronic kidney disease^[76^-^77]^, rheumatoid arthritis^[78]^, in the elderly^[79^-^80]^, and is elevated with exercise^[47]^ or a diet rich in olive oil^[81]^. Conversely, the SAA content on HDL has been found to increase in chronic kidney disease^[76^,^82]^, aging^[79]^, acute inflammation^[44^,^75]^, rheumatoid arthritis^[78]^ and cirrhosis^[73]^. Proteomic analysis of plasma specimens of AAA patients identify disease-associated reductions in HDL's major lipoproteins, namely apoA-I^[45^,^83]^ and apoA-II^[45]^. Contrasting reports, however, observe an upregulation in apoA-I and apo-J levels in this patient population^[84]^. Importantly, in several cases, changes to the HDL proteome can be observed in inflammatory or disease states without a change in total plasma HDL-C^[44^,^79]^, again highlighting the importance of looking beyond HDL-C when considering lipoprotein function in the etiology of disease. Other alterations to the HDL proteome that have been observed in vascular and inflammatory pathologies include reduced or elevated apoA-I, apoA-II, apoC-II and apoE, and elevated complement C3 and apoC-III^[44^,^73^,^75^-^76^,^79^,^82^,^85^-^86]^.

The HDL proteome, and by implication HDL function, is also subject to change by hypolipidemic agents. Green *et al*.^[87]^ report that CAD-associated changes in the HDL3 apolipoprotein profile, including increased levels of apoE coupled with decreased levels of apoF and phospholipid transfer protein, are reversed by combination therapy of atorvastatin and niacin. Niacin is also shown in a separate study to exhibit a synergistic enhancement of apoA-I in concert with atorvastatin^[70]^. Fibrates increase apoA-I levels and, to a greater extent, boost apoA-II levels^[88]^. The recent study by Gordon *et al*.^[89]^ reports that rosuvastatin dramatically increases the levels of alpha-1-antitrypsin in the large HDL fraction which in turn enhances HDL's anti-inflammatory properties. Additionally, lipid lowering drugs also alter the activity of HDL-associated anti-oxidant proteins such as PON-1 to augment its vasoprotective function^[65]^.

The HDL lipidome is another field of emerging interest. While most of the work thus far has investigated the lipidome of HDL from healthy subjects, it is becoming clear that changes to the proportions of HDL lipids in disease can have functional consequences. For example, the CEC, antioxidant, and anti-inflammatory activities of HDL are impaired with excess triglyceride, cholesteryl ester, oxidized lipids, and sphingomyelin^[90]^. A well-studied bioactive lipid on HDL is S1P, which is well known to be at least partially responsible for the anti-inflammatory actions of HDL. S1P on HDL can be reduced in CVD^[91]^ and acute inflammation^[75]^ resulting in impaired signaling to eNOS. In type 2 diabetes mellitus (T2DM), S1P has been observed to be elevated on HDL possibly as a compensatory mechanism^[92]^. Many other changes to the HDL lipidome have been observed including changes to triglyceride, phospholipid, and sphingomyelin content (***Table 1***).

Among other cargo carried on HDL are small non-coding RNAs including tRNA-derived RNA fragments, RNase P-derived RNA fragments, and microRNA (miRNA)^[93]^. MiRNAs in particular have emerged as an exciting topic in lipid research for their potential as biomarkers and in therapeutic approaches. HDL has been found to be regulated by and to carry a number of miRNAs that vary between individuals according to a number of factors including diet^[94]^, weight loss^[95]^, and CAD^[72]^. As with changes to the HDL proteome, changes to the miRNA profile of HDL can be observed even when there is no change in total plasma HDL-C^[72^,^95]^ or apoA-I levels^[94]^. Interestingly, HDL-associated miR-223, which can be altered with diet or weight loss^[94^-^95]^, has even been found to be transferred to endothelial cells^[96]^ and to alter gene expression of intercellular adhesion molecule-1 in those cells^[97]^.

HDL also carries a number of other nonpolar molecules including fat-soluble vitamins, vitamin binding proteins, carotenoids, steroids and other hormones, potentially serving as a transporter for delivery to other tissues^[93]^. Polar metabolites have also been found on HDL, some of which correlate with the insulin resistance^[98]^. An additional molecule of interest on HDL is symmetric dimethylarginine, a metabolite that is increased in children with chronic kidney disease and may be partially responsible for impaired vasoprotective actions of HDL in these patients^[99]^.

A further layer of complexity to HDL heterogeneity includes modifications to its protein components including the addition of aldehydes such as acrolein^[100]^, modifications of apoA-I by myeloperoxidase^[101]^, or carbamylation of HDL-associated proteins^[77]^. These protein modifications are associated with CVD and compromise HDL functions including cholesterol efflux, antioxidant properties, and promotion of endothelial cell migration and proliferation^[77^,^100^-^101]^.

## Cerebral vessel disease and dementia

The brain comprises only 2% of total body mass but consumes approximately 12% of total cardiac output^[102]^. The intimate association of neurons with vessels via neurovascular coupling regulates cerebral blood flow (CBF) in response to changes in neuronal activity. This coupling also maintains the necessary influx of oxygen, glucose and ions balanced by homeostatic clearance of neurotoxic by-products from the brain throughout the lifespan. As the brain cannot always easily or quickly compensate for restricted blood supply, structural and functional impairments in the cerebrovasculature can profoundly impact brain function. Central nervous system (CNS) microvessels are distinguished by the presence of the blood brain barrier (BBB), which stringently controls the movement of solutes into the brain to maintain a CNS ionic environment optimal for neuronal activity.

Cerebral vessel disease (CeVD) is one of the most common vascular pathologies of the aging brain with heterogeneous changes that undermine the integrity and function of cerebral vessels (arteries, arterioles, venules and capillaries) including atherosclerosis, arteriosclerosis, lipohyalinosis, and cerebral amyloid angiopathy (CAA)^[103^-^104]^. Increased by CVD risk factors^[105]^, CeVD can restrict CBF causing local or global ischemia in the brain through narrowing of the vessel lumen, arterial occlusion, loss of cerebrovascular resistance and micro and macro hemorrhage. Resulting brain damage from this process can lead to vascular cognitive impairment or vascular dementia (VaD), which is clinically identified by impaired locomotor function in addition to memory loss and executive dysfunction^[104]^. In particular, cerebral small vessel disease (CSVD), which manifests as white matter lesions (i.e. lacunes, lacunar infarcts, and leukoaraiosis), is a leading contributor to VaD^[106]^. How impaired cerebrovascular function may relate to cognitive decline and dementia is an area of intense interest.

Alzheimer's disease (AD), the most common form of dementia^[107]^, is clinically characterized by memory loss and conclusively diagnosed by the presence of β-amyloid (Aβ) plaques and neurofibrillary tangles in brain tissue^[108]^. The amyloid hypothesis of AD, which proposes that accumulation of Aβ aggregates is the primary pathogenic factor that initiates and drives neurodegeneration in AD, was founded on the discovery of genetic mutations that cause aberrant overproduction of Aβ in familial early onset AD (EOAD) (<65 years old) and evidence that Aβ is neurotoxic^[108]^. However, as only 1-3% of AD cases are attributed to causal mutations^[109]^ and Aβ aggregates can be present in elderly people who show no signs of cognitive decline^[110]^, major efforts are being deployed to understand the etiology of sporadic or late onset AD (LOAD) (>65 years old). Cardiovascular risk factors including hypertension, T2DM and mid-life dyslipidemia are all associated with increased AD risk^[111]^. The majority of AD patients possess extensive damage to their cerebral blood vessels^[112]^ and exhibit mixed vascular pathology with CeVD (atherosclerosis of the circle of Willis and its branches) as well as cerebrovascular lesions including leukoaraiosis, and lacunar infarcts, microbleeds, microinfarcts, and CAA^[113]^. Importantly, increased severity of CeVD in subjects over 65 years of age is associated with lower scores across several cognitive domains including episodic memory and perceptual speed, the respective neuropsychological hallmarks of AD and VaD, which remain even after adjusting for established genetic AD or vascular risk factors^[114]^. Intriguingly, white matter hyperintensities indicative of CSVD were recently found to be elevated in EOAD mutation carriers (mean age 39 years) that were detectable at least 6 years prior to clinical onset^[115]^. That these EOAD subjects were too young to exhibit classical age-related cardiovascular risk factors provides compelling support that cerebrovascular dysfunction may be an important driver of neuronal compromise and cognitive decline.

The brains of AD patients show a plethora of structural and functional vascular abnormalities that correlate with severity of neurodegeneration, including reduced microvascular density with remaining vessels appearing tortuous and string-like^[112^,^116]^. One potential cause of this brain vessel atrophy may be the Aβ-driven vascular pathology of CAA, in which Aβ is deposited in the walls of the arteries, arterioles and capillaries in the leptomeninges and cerebral cortex^[117]^. CAA prevalence in AD is 80-90%^[118]^ and CAA may contribute to degeneration of mural cells in arteries, arterioles, and capillaries, leading to vessel stiffening and impaired vasomotor function^[117]^. Notably, weakening of cerebral vessels increases their susceptibility to rupture and CAA is also associated with ischemic lesions, micro- and macro-hemorrhages, and impaired CBF^[118]^. Microscopic cerebral hemorrhages, also known as cerebral microbleeds (CMBs) are a type of CSVD that indicates weakness within the microvascular system^[119]^. Studies show that CAA-associated vasculopathies lead to the development of CMBs in the lobar temporal and parietal cortex although there is some debate as to the direct association between CAA and CMBs^[120]^. In contrast, CMBs in non-lobar deep white matter regions are associated with vascular risk factors such as stroke and hypertension^[121]^. Recent findings from a prospective analysis of the Rotterdam cohort suggest CMBs may prove to be useful predictors of future cognitive decline and pre-clinical dementia regardless of their location in the brain^[122]^. Whether microhemorrhages affect cognitive status and are a reliable biomarker of cognitive decline is a key debate, with one study reporting no effect of CMBs on cognitive function^[123]^ contrasting with another study that found an association between frontal lobe lacunar infarcts and pre-dementia^[124]^.

The close relationship between AD and cerebral vascular pathology raises the hypothesis that vascular damage may plays a considerable role in precipitating and driving AD pathogenesis. The two-hit vascular hypothesis proposes that vascular risk factors (hit one) leads to the cerebrovascular dysfunction (i.e. BBB dysfunction, oligaemia) that precedes cognitive impairment^[116]^. This vascular damage induces early neuronal dysfunction due to the accumulation of neurotoxic molecules, capillary hypoperfusion and altered Aβ metabolism that accelerates Aβ retention and accumulation in the brain^[116]^. Increased cerebral Aβ represents hit two, which amplifies neuronal dysfunction leading to a self-propagating acceleration of neurodegeneration, cognitive decline and ultimately dementia^[116]^.

VaD is the second most common dementia^[125]^, wherein, unlike AD, various types of vascular injury including ischemic, hemorrhagic, or hypoperfusion directly causes cognitive impairment. It is increasingly appreciated that AD and VaD share considerable overlap in clinical^[126]^, pathological^[103]^ and epidemiological features^[127]^. Interestingly, up to 45% of clinical dementia cases have evidence of mixed neuropathology for both AD and VaD^[128]^ and the prevalence of mixed dementia (AD and VaD) increases with age^[129]^. This lends support to the argument that vascular dysfunction interacts synergistically with other pathogenic neurodegenerative pathways to promote various forms of dementia. Mirroring the worsening cognitive decline seen in humans exhibiting cerebral hypoperfusion^[103]^, experimental restriction of CBF in animals recapitulates both the amyloid and vascular neuropathology of mixed dementia^[130]^. Chronic cerebral hypoperfusion as a result of cerebrovascular dysfunction may therefore serve as a common catalyst for the development of CAA and subsequent Aβ-associated pathologies^[130]^. Stroke is an established risk factor for AD^[105]^, and while an initial report of increased cerebral Aβ levels in ischemic stroke patients^[131]^ failed to reproduce in a subsequent larger cohort^[132]^, new evidence that cerebral hypoxia may diminish enzymatic Aβ-degradation^[133]^ offers a potential mechanism by which cerebrovascular incidents may accelerate AD pathogenesis.

An important point for potential therapeutic considerations is that cerebrovascular lesions may correlate with more severe cognitive dysfunction in early AD rather than late in progression^[103]^. As 20 years of research on the amyloid hypothesis and Aβ- targeting therapies have not yet produced an approved treatment for AD, it is imperative that the multifactorial aspects of AD be addressed in the future. Given the beneficial vasoprotective roles of HDL in peripheral vessels, expanding HDL research toward the cerebrovasculature and neurodegeneration may be highly promising.

## Lipoproteins and cognitive function

The brain is the most cholesterol-rich reservoir in the body, containing 25% of the body's total cholesterol content^[134]^. The connection of lipid metabolism to AD was first noted in Dr. Alois Alzheimer's characterization of the disease in 1906 that described lipid deposits in the brain^[135]^. Today, genome wide association studies confirm this connection, with the identification of confirmed AD risk genes that function in various aspects of lipid metabolism^[136]^. Of these, genetic variation in apoE is the strongest genetic risk factor for AD in humans, with *APOE4* ^[137^-^138]^, *APOE3* neutral^[139]^, and *APOE2* protective^[140]^. Over 60% of AD cases possess at least one *APOE4* allele^[141]^, and carriers of the *APOE4* allele show increased risk, earlier onset, and exacerbated cognitive decline^[137^-^138^,^141]^. Despite this strong association, the exact mechanisms by which apoE, which is produced by both astrocytes and microglia, modifies AD risk in an isoform-specific manner are not yet completely defined. One undisputed function of apoE relates to its role in Aβ deposition, as *APOE4* carriers consistently develop greater Aβ burden at an earlier age compared to non-*APOE4* carriers^[142^–^146]^. *APOE4* is the strongest genetic risk factor of LOAD^[137^,^147]^, and moreover predisposes carriers to cardiovascular disease^[148]^, reinforcing the importance of cholesterol metabolism in the vascular-mediated pathogenesis of sporadic LOAD. Importantly, apoE may also impair cerebrovascular function, as apoE4 is associated with aberrant binding and cell signaling at the neurovascular unit resulting in diminished Aβ clearance^[149^-^150]^, reduced eNOS expression^[151]^, vascular inflammation, and BBB dysfunction^[152]^. Exacerbation of vascular dysfunction by apoE4 would be expected to aggravate cognitive decline in *APOE4* carriers, and while there is a suggestion of such a relationship^[153]^, others find a lack of association of *APOE4* and cognitive impairment with concurrent CeVD^[154]^. How *APOE4* may play a multifaceted role in AD pathogenesis remains to be fully elucidated.

Apo J, or clusterin, is the other major lipoprotein besides apoE that is abundantly produced within the CNS^[155]^. Genetic mutations in the clusterin gene (CLU) were identified by two independent GWAS studies as risk factors for LOAD^[156^-^157]^. Clusterin is elevated in AD brains^[158]^, present within Aβ plaques^[158^-^159]^ and co-localizes with Aβ deposits in CAA-affected leptomenigeal arteries^[160]^. Clusterin has been shown to facilitate Aβ egress from the brain when co-injected into mice^[161]^. Recent findings by Miners *et al*.^[162]^ also support a role for clusterin in regional Aβ clearance in humans. Intriguingly, they showed that although clusterin levels are highest in brain regions with plaque pathology, the molar ratio of clusterin: Aβ42 surprisingly declines with insoluble Aβ42 levels in a region-dependent manner, suggesting that rising Aβ42 levels outstrip increased clusterin levels, thereby decreasing Aβ clearance and promoting its region-specific deposition. In vitro studies suggest that clusterin acts as a chaperone protein facilitating Aβ egress at the BBB^[161^,^163]^ and Aβ transport to microglia for degradation via interactions with the microglial receptor TREM-2^[164]^. Clusterin also interferes with Aβ peptide aggregation and neutralizes Aβ oligomer neurotoxicity^[165]^, and deficiency of clusterin signaling through its receptor, plexin A4, leads to memory and learning deficits^[166]^. Clusterin has also been linked to accelerated atrophy in brain regions first affected by AD through an unknown interaction with Aβ^[167]^. Further work is needed to determine the dynamics of this glycoprotein in the context of health and neurodegeneration.

In addition to brain-derived apoE and apoJ, circulating lipoproteins may also be important to cerebrovascular health. Along with its defined association with CVD, high levels of plasma HDL-C in elderly people are associated with better memory^[168^-^169]^, lower Aβ burden^[170]^, and less cognitive decline^[171]^. Conversely, HDL-C has been found to be reduced in AD subjects who have vascular risk factors^[172]^, and plasma apoA-I has been reported to be reduced in AD patients^[173]^ and negatively associates with cognitive decline independent of Aβ, indicating a protective homeostatic role for apoA-I against cognitive decline in the elderly^[174]^. In symptomatic AD patients, plasma apoA-I levels negatively correlate with hippocampal and whole brain volume as well as mean entorhinal cortical thickness^[175]^. Compared to age-matched cognitively healthy controls, levels of cerebrospinal fluid (CSF) apoA-I increase during aging but are significantly lower in AD and mild cognitive impairment patients compared to age-matched cognitively normal controls^[176]^. Additionally, AD patients were found to have significantly lower gene expression of *APOA1*, *APOC3* and *APOA4*, which correlated with AD severity^[177]^. However, controversy exists in the relationship between HDL-C levels and cognition, as other studies report no relationship between HDL-C and dementia^[178]^ nor a link between genetically altered HDL-C and AD using Mendelian randomization approaches^[179^-^180]^. In a Japanese cohort, the previously reported positive associations of *APOA1* polymorphisms and AD^[181^-^182]^ were not reproduced, but a single nucleotide polymorphism, Rs7659 in apoD, was correlated to EOAD after stratification against *APOE4* genotype^[183]^. The discovery that methylation of an *APOA1* CpG site increases protein levels of plasma apoA-I and negatively correlates with episodic memory in an older population again suggests a complex relationship between HDL and cognition^[184]^. Notably, this study identifies epigenetic regulation of cholesterol metabolism and the impact of environmental and lifestyle influences as new areas of interest in dementia research. As HDL functions can be discordant with HDL-C levels, the usefulness of HDL-C as a parameter of HDL effectiveness in CNS disorders remains to be determined. On the other hand, changes in HDL's functional antioxidant activity, as estimated by reduced activity of serum PON-1, correlates with cognitive decline particularly in mixed AD-VaD dementia^[185^-^186]^.

Associations of HDL reach beyond AD to include other neurodegenerative diseases. A protective association of HDL has been found in multiple sclerosis in which HDL-C inversely correlates with BBB damage and leukocyte extravasation^[187]^. Similarly, in Parkinson's Disease, plasma HDL-C levels are reduced especially in the early stage of the disease^[188^–^190]^. Intriguingly, HDL-C levels and RCT function are only diminished in cases of AD with cardiovascular co-morbidity when compared to AD without co-cardiovascular morbidities or to age-matched, healthy cognitively normal controls^[172]^.

## HDL and dementia: mechanisms and therapeutic potential

Mechanisms by which circulating HDL affects the CNS likely involve the cerebral vasculature, particularly with respect to CAA and inflammation. Studies in experimental models allow such mechanisms to be explored. For example, genetic deletion of apoA-I in AD mice^[191]^ worsens CAA and neuroinflammation and exacerbates cognitive function without an overall change in parenchymal amyloid. In harmony with this finding, transgenic overexpression of human apoA-I from its endogenous promoter that drives expression in liver and intestine in AD mice selectively ameliorates CAA and neuroinflammation and partially restores memory^[192]^. More recently, intravenous injection of reconstituted human HDL into AD mice was found to acutely reduce soluble amyloid levels in the brain^[193]^, consistent with a previous rodent study in which oral administration of an apoA-I mimetic reduces Aβ burden in the brain^[194]^. As apoA-I is not synthesized in the brain by glial or neuronal cells yet is present in the CSF at levels similar to that of its brain-derived apoE counterpart^[195]^, and lipid-poor plasma apoA-I gains accesses to the CSF via the choroid plexus in mice^[196]^, an emerging question is whether apoA-I may affect cerebral vessels from the “blood side” or “brain side.” This question has important implications for possible therapeutic opportunities that could involve systemically acting agents that do not necessarily need to cross the BBB. Equally importantly, such therapeutic options could leverage on the considerable investments already made to develop cardiovascular therapies. Both in vitro and in vivo pre-clinical studies report that administration of plasma-isolated human HDL after cerebrovascular insult improves BBB integrity^[197]^ and limits neuroinflammation by inhibiting neutrophil extravasation into the brain^[198^-^199]^, offering one explanation of the observed preservation of cognition post-stroke with this HDL therapy. Conclusive investigation into whether these improvements in brain microvasculature directly translate into cognitive enhancement or stabilization has yet to be thoroughly explored and warrants further research.

While several epidemiological and animal model studies suggest that statins may protect from dementia, comprehensive analysis of randomized clinical trials have found no beneficial effect when statin use was initiated in late life^[200^-^201]^. Studies in the Taiwanese population suggest the time of drug administration, drug dosage and duration are important factors for statins to affect dementia outcome. Lin *et al.* found that use of statins prior to definite AD diagnosis associates with delayed disease progression in mild-moderate AD patients^[202]^. Chen *et al.* observed that dementia risk is decreased by high dose and long-term use of statins, an effect that is not observed with fibrates or other lipid-lowering drugs (acipimox, cholestyramine, niceritrol, nicofuranose, nicomol, and probucol^[203]^. Conclusively determining whether statins are effective for delaying or treating dementia will require further attention to the timing, dosage and duration of statin use. Fibrates, another class of lipid-lowering agents in clinical use, also fails to show any benefit to prevent cognitive decline in older populations^[204]^ and those at risk for CVD^[205]^. Whether dementia risk or progression may be affected by cardiovascular therapies that alter circulating lipoprotein levels and functions, including fibrates, niacin, CETP inhibitors and the recently released PCSK9 inhibitors^[206]^, has yet to be systematically tested. Despite the controversial benefit of statins supplemented with niacin on CAD^[207]^, the effect of combination treatments on cognition is still relatively unknown and may prove to be a more potent option to limit dementia risk.

Considerable investment has been made in testing pharmacological agents that affect Liver-X-Receptor (LXR), Retinoid-X-Receptor (RXR) and peroxisome proliferator-activated receptor gamma pathways, as these are master regulators of both lipid metabolism and inflammation^[208]^ and influence key pathogenic pathways in neurodegenerative disease. *ABCA1*, a downstream gene target of these nuclear receptor pathways, has been consistently and independently shown to protect against AD phenotypes in animals^[209^–^212]^ and an epidemiological study in Denmark identified a loss of function *ABCA1* gene as a risk factor for both AD and cerebrovascular disease^[213]^. In pre-clinical studies pharmacological activation of LXR/RXR effectively ameliorates AD phenotypes, although with varying changes in cerebral Aβ pathology^[214^–^221]^. There is mounting evidence in both AD and experimental stroke models that LXR/RXR agonists mediate improvements in cerebrovascular health via preservation of BBB integrity, which is one potential mechanism by which this drug class may exert neurological and cognitive benefits^[222^–^227]^. Efficacy of LXR/RXR agonists in ameliorating AD pathology and memory loss in mice is dependent on ABCA1^[228]^, and although not a direct gene target of the LXRs, CSF levels of apoA-I are increased with an oral regimen of an LXR agonist^[229]^. Further studies are needed to delineate the contribution of peripheral lipoproteins as mediators of the protective action of LXR agonists against neurodegeneration.

Bexarotene, a USA Food and Drug Administration approved anti-cancer drug and RXR agonist, was reported to rapidly decrease Aβ pathology and significantly ameliorate cognitive decline in AD mice^[230]^. This study spearheaded several investigations into the potential therapeutic benefit of bexarotene, which is currently being examined in AD clinical trials despite mixed data on its effectiveness in AD animal models^[218^,^231^–^236]^. The 2016 phase 2a clinical trial in which bexarotene was administered over four weeks to early AD patients failed to reduce brain amyloid levels as measured by positron emission tomography^[237]^. Although post-hoc analysis suggests potential effects in non-*APOE4* carriers^[237]^, larger numbers will be needed to decisively determine the efficacy of bexarotene in subjects of each *APOE* genotype. A case report of improved cognition in an AD patient with no concurrent changes in Aβ neuropathology leaves room for cautious optimism^[238]^. A major drawback to further development of LXR/RXR agonists is their undesirable side effects, namely hyperlipidemia caused by increased fatty acid synthesis in the liver^[208]^. New evidence that statins may interfere with ABCA1 expression^[70^,^239]^ may also necessitate the consideration of alternative cholesterol management plans to traditional statin use.

## Gaps and opportunities

Taken together, much remains to be discovered about HDL's role in health and disease. In particular, despite its association with dementia, the mechanisms by which HDL may promote healthy aging and protect from neurodegeneration remain far from clear. Studies investigating the role of HDL in ameliorating cerebral vessel disease could be expanded to include cognitive aspects. Other unanswered questions include what compositional changes of HDL occur throughout neurodegeneration and dementia and how these compare to other chronic inflammatory states such as in metabolic syndrome, T2DM, chronic kidney disease, or CVD. Whether dementia-specific compositional changes of HDL may impair CNS function and potentially lead to new therapeutic targets remains to be determined. Filling these knowledge gaps will improve our understanding of the dynamic nature of HDL for vascular physiology and neurodegeneration.
